# Dietary Intake and Anthropometric Status Differ for Anaemic and Non-anaemic Rural South African Infants Aged 6–12 Months

**Published:** 2007-09

**Authors:** Mieke Faber

**Affiliations:** Nutritional Intervention Research Unit, Medical Research Council, South Africa

**Keywords:** Anaemia, Iron-deficiency, Anthropometry, Haemoglobin, Infant growth, Infants, Risk factors, South Africa

## Abstract

The study was undertaken to determine anthropometric measurements, sociodemographic data, and dietary intake of 238 anaemic and 241 non-anaemic rural South African infants aged 6–12 months. Logistic regression with anaemia as a dependent variable showed an odds ratio (OR) of 1.89 (95% confidence interval [CI] 1.01–3.52) for low birth-weight, 2.04 (CI 1.29–3.22) for maternal age 20 years or younger, 2.21 (CI 1.29–3.76) for consumption of tea, and 0.40 (CI 0.26–0.63) for formula feeding. The anaemic infants, aged 6−<9 months, had a lower average weight gain per month than the non-anaemic infants (727 g vs 772 g; p=0.040, analysis of variance). Logistic regression with underweight as a dependent variable showed an OR of 3.55 (CI 1.26–10.01) for anaemia, and with stunting as a dependent variable, the OR was 2.71 (CI 1.46–5.02). Low birth-weight, a young mother aged 20 years or younger, and consumption of tea were identified as risk factors for anaemia, while formula feeding was shown to have a protective effect. The anaemic infants were more likely to show growth faltering.

## INTRODUCTION

Iron deficiency is one of the most prevalent nutrient deficiencies in the world. Although iron deficiency may occur throughout the lifespan, women and young children are the most commonly and severely affected (1). Full-term infants normally have adequate iron stores at birth. After the age of 4–6 months, their iron stores become depleted, making them vulnerable to iron deficiency and finally iron-deficiency anaemia as reflected in low haemoglobin concentrations. De Pee *et al* reported that iron stores might become depleted even earlier, at 3–5 months (2). Infants have high requirements of iron because of rapid growth, and during the second half of infancy, the diet must supply considerable amounts of iron as the infant can no longer rely on iron stores to meet the requirements of iron. Low haemoglobin during infancy impairs child development; an association between haemoglobin and measures of cognitive and motor development has been found in several correlation and case-control studies (3). The iron status of infants is influenced by dietary (e.g. amount and type of iron consumed, and the presence of inhibitors and enhancers of iron absorption), physiological (e.g. birth-weight) and environmental (e.g. sociodemographic background) factors. The identification of risk factors for anaemia in particular settings is an important step in the development of appropriate and effective nutrition programmes.

Two surveys of South African infants, aged 6–12 months, showed that the proportion of infants with haemoglobin concentrations below 110 g/L ranged from 40% to 83% (4,5). The baseline survey of a randomized controlled trial of South African infants, aged 6–12 months, in a rural area of low socioeconomic status showed that 49% of 498 infants had haemoglobin concentration below 110 g/L (unpublished data), which is similar to the national figure for infants in this age-category (6). The aim of the present study was to determine the anthropometric status and identify risk factors associated with anaemia of rural South African infants aged 6–12 months, using the findings of the above-mentioned cross-sectional baseline survey.

## MATERIALS AND METHODS

Participants resided in The Valley of a Thousand Hills, a rural area in KwaZulu-Natal province, South Africa. The population density is low as families are scattered over a large mountainous area. The community is predominantly Zulu-speaking. This study was part of the baseline survey of a randomized controlled trial that was approved by the Ethics Committee of the South African Medical Research Council. Written informed consents were obtained from mothers before data collection.

All 6–12-month old infants in the catchment area of eight community health centres were recruited through the community health worker programme of The Valley Trust, a non-governmental organization. The community health worker programme covered all children in the catchment area of the health centres. Experienced fieldworkers interviewed the mother or caregiver (a member of the family, usually the grandmother of the child, in whose care the child was during the day), hereafter collectively referred to as ‘caregivers’ in the local language. Information on sociodemographics, breastfeeding, and complementary feeding was collected using a questionnaire that was developed following the guidelines of Gross *et al.* (7). An unquantified food-frequency questionnaire was used for determining the frequency of usually-consumed food items. Data from previous studies were used for compiling a list of food items for which the frequency of consumption for the past month was recorded with the respondents having a choice of five options, namely (a) every day, (b) most days (not every day, but at least 4 days per week), (c) approximately once a week (less than 4 days per week, but at least once per week), (d) seldom (less often than once a week), and (e) never. The questionnaires were piloted and revised before being finalized.

Nutrient intake was determined using a quantified single 24-hour dietary recall. Fresh food, plastic food models, household utensils, and three-dimensional sponge models were used for quantifying and recording food consumption of the previous day. In addition, dry oats were used for quantifying portion sizes of certain food items, especially cooked food. The caregiver used the dry oats for indicating the quantity resembling the amount of food that the infant consumed, which the fieldworker then quantified using a measuring cup. Food intakes reported in household measures were converted into weight using the MRC Food Quantities Manual (8). An intake of 675 mL and 615 mL of breastmilk was assumed for infants aged 6-<9 and 9-<12 months respectively (9). For infants who consumed both breastmilk and formula milk, the volume of the formula milk was subtracted from 675 mL and 615 mL—depending on the age of the infant—to obtain an estimate for the quantity of breastmilk consumed. The SAS software package was used for converting food intakes to macro- and micronutrients, using the MRC Food Composition Tables (10) as the food database. Two indicators for dietary diversity were calculated, namely (a) the number of food items and (b) the number of food groups reported for the dietary recall period. Foods were grouped into 11 groups: milk and milk products; infant foods; eggs; fish; meat and meat products; legumes; cereals; fats; fruits; vegetables; and sugars.

Anthropometric measurements were taken with the infant in light clothing. Weight was measured on a calibrated load cell-operated digital scale (UC-300 Precision Health Scale, Mascot, Tokyo, Japan) accurate to 50 g. Recumbent length was measured to the nearest 0.1 cm using a length board with a fixed headboard and a sliding footboard. Date of birth and birth-weight were obtained from the child's clinic card. Birth-length was not recorded as this information was incomplete on most clinic cards. The child's growth rate (g/month) was calculated based on the following formula:


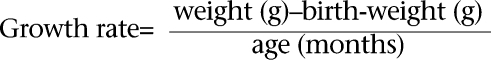


The anthropometric data and age of the child were used for yielding three measures of nutritional status, namely length-for-age, weight-for-age, and weight-for-length, which were expressed as z-scores using the Epi Info 2000 software package. Children with length-for-age z-score, weight-for-age z-score, and weight-for-length z-score below −2 SD of the median of the reference population were classified as stunted, underweight, and wasted respectively.

A blood sample was obtained by antecubital venapuncture. One mL of blood was transferred to an EDTA tube. Haemoglobin was determined on the day of blood collection by the cyanomethaemoglobin method (Drabkin reagent) using a portable photometer (Ames Minilab, Product no. 7316; Miles Inc., IN, USA). Blood samples of known haemoglobin concentrations were used as external quality control; the values measured for these quality-control samples were 113 g/L (vs the known value of 112 g/L) and 163 g/L (vs the known value of 161 g/L). The coefficient of variation for measurements of haemoglobin was below 5%. Anaemia was defined as a haemoglobin concentration below 110 g/L (11).

### Data analysis

The SPSS software (version 10.0) (SPSS, Inc., Chicago) was used for data analysis, except for the 24-hour dietary recall data for which the SAS software (version 8.2) (SAS Institute Inc., Cary, NC) was used. The differences between anaemic and non-anaemic infants were determined using the chi-square test (categorical data), analysis of variance (continuous data), and Wilcoxon two-sample test (quantified dietary intake). Potential dietary, physiological and environmental risk factors that were shown to differ between the two groups were then used for multiple logistic regression analysis, which included the potential risk factors as the independent variables and haemoglobin status (anaemic or non-anaemic) as the dependent variable. Nutrient intake was not included in the multiple logistic regression analysis because a single 24-hour dietary recall is not reflective of the individual's dietary intake (it is a reflection of the average intake of a group). Logistic regression analysis was also done with anthropometric status as a dependent variable and anaemia as an independent variable. The odds ratios (ORs) and 95% confidence intervals (CIs) were estimated for the regression parameters. A p value of <0.05 was considered statistically significant.

## RESULTS

In total, 479 infants (250 boys and 229 girls), aged 6–12 months, were included in the study. Infants were categorized as either anaemic (haemoglobin <110 g/L; n=238) or non-anaemic (haemoglobin ≥110 g/L; n=241). Household demographic characteristics, age, anthropometric indices, and type of milk feeding of the anaemic and non-anaemic infants are given in Table 1. The average age was 9.0±2.1 months for both the groups.

**Table 1:** Household demographic characteristics, age, anthropometric indices, and type of milk-feeding to anaemic and non-anaemic infants aged 6–12 months

Characteristics	Anaemic (n=238)	Non-anaemic (n=241)	p value∗
Access to tap water (%)	94	95	NS
Access to toilet facilities (%)	89	92	NS
Access to electricity (%)	72	84	0.001
Size of household size			
Mean (SD)	9 (4)	9 (4)	NS
Age (years) of mother			
Mean (SD)	24.3 (6.8)	26.5 (6.8)	0.0001
20 years or younger (%)	40	23	0.0001
Age (months) of child			
Mean (SD)	9.0 (2.1)	9.0 (2.1)	NS
Gender (%)			
Boys	54	50	NS
Girls	46	50	
Birth-weight (g)			
Mean (SD)	2,935 (554)	3,050 (501)	0.029
Low birth-weight† (%)	17	9	0.001
Length-for-age z-score			
Mean (SD)	-1.26 (1.25)	-0.78 (0.99)	0.0001
Stunted‡ (%)	23	9	0.0001
Weight-for-age z-score			
Mean (SD)	−0.12 (1.39)	0.47 (1.29)	0.0001
Under-weight¶ (%)	10	3	0.001
Weight-for-height z-score			
Mean (SD)	1.05 (1.11)	1.36 (1.21)	0.004
Wasted §(%)	0	0	–
Overweight∗∗ (%)	18	30	0.001
Growth rate (g/month)‡‡			
mean (SD)			
6–<9 months	727 (161)	772 (146)	0.040
9–12 months	599 (133)	610 (120)	NS
Breastfeeding initiated (%)	97	97	NS
Duration (months) of exclusive breastfeeding (%)			
1	74	72	NS
2	66	63	
3	52	52	
4	24	25	
5	10	12	
6	7	8	
Current breastfeeding (%)	86	77	0.012
Current formula feeding (%)	26	48	0.0001
Age of introduction of solid foods			
Mean (SD)	3.3 (1.4)	3.4 (1.5)	NS

∗ Between group difference: chi-square test (categorical data) and analysis of variance (continuous data);

† Below 2,500 g;

‡ Length-for-age z-score below −2 SD of the median of the reference population;

¶ Weight-for-age z-score below −2 SD of the median of the reference population; Weight-for-length z-score below −2 SD of the median of the reference population;

∗∗ Weight-for-length z-score above 2 SD of the median of the reference population;

‡‡ Growth rate=(weight-birth-weight)/age NS=Not significant; SD=Standard deviation

Household demographic characteristics were similar between the two groups, except for availability of electricity within the household (anaemic group 72%, non-anaemic group 84%; p=0.001). Approximately 90% or more of the households in both the groups had access to tap-water and toilet facilities. Mothers of the anaemic infants were younger than mothers of the non-anaemic infants (24.3±6.8 vs 26.5±6.8 years; p=0.0001). At the time of the survey, 32% of the mothers were aged 20 years or younger (anaemic group 40%, non-anaemic group 23%; p=0.0001).

Birth-weights were not available for 40 infants who were born at home. Of the 439 infants who were born in a health facility, birth-weights were obtained from the clinic cards for 402 infants (the clinic cards for the remaining 37 infants born in a health facility were incomplete as the birth-weight was not recorded). Thirteen percent of these infants had a low birth-weight (<2,500 g), with more anaemic than non-anaemic infants having low birth-weight (17% vs 9%; p=0.001). The mean z-score values for length-for-age of both the groups were negative, indicating a shift towards a slowing in linear growth according to the international criteria. Linear growth retardation (stunting) was more severe among the anaemic than among the non-anaemic infants (23% vs 9%; p=0.0001). The anaemic infants had a higher prevalence of under-weight (10% vs 3%; p=0.001). For younger infants (6-<9 months), the average weight gain per month was lower for the anaemic than for the non-anaemic infants (727 g vs 772 g; p=0.040).

Breastfeeding had been initiated for 96% of the infants; 80% of the infants were breastfeeding at the time of the survey; exclusive breastfeeding up to the age of six months was not practised. The type of milk-feeding differed between the anaemic and the non-anaemic infants. At the time of the survey, 86% of the anaemic infants were breastfeeding (vs 77% of the non-anaemic infants), and 25% were receiving formula milk (vs 48% of the non-anaemic infants). Solid foods were introduced at an average age of 3.3±1.5 months, with no difference between the anaemic and the non-anaemic infants.

The proportion of infants who usually consumed certain food items at least four days per week was determined using the data from the unquantified food-frequency questionnaire and is presented in Table 2. Yoghurt (anaemic infants 17%, non-anaemic infants 27%; p=0.028), fortified infant-cereals (anaemic infants 43%, non-anaemic infants 60%; p=0.006), and consumption of tea (anaemic infants 29%, non-anaemic infants 17%; p=0.034) were the only food items that differed between the two groups.

**Table 2:** Proportion of anaemic and non-anaemic infants, aged 6–12 months, who usually consumed foods at least 4 days per week as determined using an unquantified food-frequency questionnaire

Food	Anaemic infants (n=238)	Non-anaemic infants (n=241)	p value
Cereals/starches
Bread	45	44	NS
Maize–meal porridge–soft	90	86	NS
Maize–meal porridge–stiff	30	21	NS
Maize–meal porridge–fermented	3	1	NS
Infant-cereal	43	60	0.006
Rice	32	31	NS
Potato	43	45	NS
Sweet potato	0	1	NS
Dairy products			
Fresh milk	3	6	NS
Milk-powder	14	14	NS
Yoghurt	17	30	0.028
Animal foods			
Meat	30	20	NS
Chicken	9	15	NS
Fish	1	2	NS
Eggs	34	40	NS
Legumes			
Beans	1	1	NS
Soya-protein	9	9	NS
Peanut butter	24	30	NS
Vegetables			
Pumpkin	20	30	NS
Butternut	18	25	NS
Carrots	3	3	NS
Dark-green leafy vegetables	10	10	NS
Cabbage	7	5	NS
Tomato	4	5	NS
Fruits			
Apple (mostly cooked)	8	9	NS
Banana	28	29	NS
Orange	34	38	NS
Miscellaneous			
Sugar	54	46	NS
Biscuits	24	28	NS
Sweets	9	7	NS
Savoury snacks	40	44	NS
Carbonated drinks	9	15	NS
Tea	29	17	0.034

NS=Not significant

Information on quantified dietary intake was obtained for 447 infants; for the remaining infants, the caregivers could not provide reliable information as the infants were not in their care for the entire 24 hours. Table 3 shows intakes of energy and macro- and micronutrients. Energy intake did not differ between the two groups. The anaemic infants had lower intakes for protein (especially animal protein), calcium, iron, zinc, thiamine, riboflavin, niacin, vitamin B_6_, vitamin B_12_, and vitamin C than the non-anaemic infants. Iron intake was well below the estimated average requirement (EAR) of the US dietary reference intakes (15) for both the groups.

**Table 3:** Median and interquartile range for intake of energy and macro- and micronutrients by anaemic and non-anaemic infants aged 6–12 months

Type of food	AI∗	Dietary intake		
		Anaemic infants (n=227)	Non-anaemic infants (n=220)	p value†
Energy (kJ)	3,121 (boys)‡ 2,839 (girls)‡	3,424 (2,998; 3,981)	3,525 (3,031; 4,040)	NS
Protein (g)	13.5	16 (12; 22)	17 (14; 23)	0.0244
Plant protein		6 (3; 9)	6 (3; 8)	NS
Animal protein		7 (6; 13)	10 (6; 15)	0.0009
Total fat (g)	30	39 (34; 43)	38 (33; 44)	NS
Carbohydrate (g)	95	102 (87; 119)	106 (88; 131)	NS
Calcium (mg)	270	295 (236; 407)	371 (277; 506)	0.0001
Iron (mg)	6.9¶	2.2 (0.9; 4.6)	4.0 (1.6; 7.5)	0.0001
Magnesium (mg)	75	78 (56; 115)	83 (61; 111)	NS
Zinc (mg)	2.5¶	2.3 (1.8; 3.1)	2.9 (2.0; 4.1)	0.0001
Vitamin A (íg RE)	500	515 (446; 673)	537 (463; 733)	NS
Thiamin (mg)	0.3	0.35 (0.25; 0.47)	0.41 (0.30; 0.54)	0.0015
Riboflavin (mg)	0.4	0.46 (0.34; 0.74)	0.62 (0.43; 1.04)	0.0001
Niacin (mg)	4	3.1 (2.2; 4.3)	4.0 (2.9; 5.7)	0.0001
Vitamin B6 (mg)	0.3	0.32 (0.20; 0.50)	0.42 (0.28; 0.66)	0.0001	79
Vitamin B12 (μg)	0.5	0.20 (0; 0.97)	0.75 (1.10; 1.44)	0.0001
Folic acid (μg)	80	71 (50; 118)	74 (53; 109)	NS
Vitamin C (mg)	50	42 (34; 58)	77 (39; 77)	0.0001

∗ Adequate intake of the US DRI published by the Institute of Medicine (12-16);

† Between group difference: Wilcoxon two-sample test;

‡ Estimated energy requirement of the US DRI published by the Institute of Medicine;

¶ Estimated average requirement of the US DRI published by the Institute of Medicine

AI=Adequate intake; DRI=Dietary reference intakes; KJ=Kilojoule; NS=Not significant; RE=Retinol equivalents; US=United States

In total, 77 food items were reported for the 447 infants for the recall period, with an average of six food items per infant (anaemic infants 6.6±2.5; non-anaemic infants 6.6±2.5). On average, five of the possible 11 food groups were reported per infant, with no difference between the two groups (anaemic infants 4.9±1.4; non-anaemic infants 4.8±1.5).

### Multivariate analysis

A binary logistic regression model (forward entering) was developed using the potential risk factors that differed between the two groups. The model controlled for age. Being underweight or stunted was not included as these were probably not risk factors but outcomes of iron deficiency. Nutrient intake was also not included, as at least two days of dietary recording are needed to determine inadequate intake by individuals (17). Low birth-weight (p=0.046), maternal age 20 years or younger (p=0.002), and consumption of tea (p=0.004) were identified as risk factors associated with anaemia, and formula feeding (p=0.0001) had a protective effect (Table 4). When controlling for birth-weight and entering anaemia as the independent variable and growth faltering as the dependent variable into logistic regression analysis, the odds ratio (95% CI) that an anaemic infant will be underweight was 3.55 (CI 1.26–10.01) and that the anaemic infant will be stunted was 2.71 (CI 1.46–5.02).

**Table 4:** Risk factors for anaemia (haemoglobin concentration below 110 g/L) as assessed by multiple logistic regression analysis

Variable	Odds ratio	95% CI	p value
Birth-weight (g)			
≥2,500	1		
≤2,500	1.89	1.01–3.52	0.046
Formula milk			
No	1		
Yes	0.40	0.26–0.63	0.0001
Consumption of tea			
No	1		
Yes	2.21	1.29–3.76	0.004
Age (years) of mother			
>20	1		
≥20	2.04	1.29–3.22	0.002

CI=Confidence interval

## DISCUSSION

Three risk factors—low birth-weight, a young mother, and consumption of tea—were identified as risk factors associated with anaemia, while formula feeding was shown to have a protective effect. The anaemic infants were more likely to show growth faltering.

The results of the study showed that low birth-weight was a risk factor for anaemia. An association between iron status and birth-weight has been documented previously (18,19). Iron stores of newborn infants are proportional to body-weight and infants with low birth-weight have, therefore, relatively small iron stores.

Infants of young mothers were more likely to be anaemic. Similar findings were observed in Indonesia (2). Poor maternal caring capacity of the young mother and the high nutritional demands of teenage pregnancy could have contributed towards this association. The prevalence of iron deficiency is high in adolescent females (20), and anaemia during pregnancy is a risk factor for iron-deficiency anaemia in infancy (2,21). Optimal nutrition during pregnancy should be ensured for all pregnant women, but more so for teenage pregnancies.

The initiation rate of breastfeeding, the prevalence of exclusive breastfeeding, and the average age for introducing solid foods did not differ between the anaemic and the non-anaemic infants. Current breastfeeding and formula feeding, however, differed between the two groups.

The iron in breastmilk is highly bioavailable, but the concentration is very low. In this study, 59% of the 278 infants who received breastmilk but no other milk-feeds at the time of the survey were anaemic. Logistic regression analysis showed that formula feeding had a protective effect on iron status. After the age of six months, nearly all of the infants’ iron requirements must be supplied by the complementary diet, which, in this study population, was inadequate (22). It has been recognized that it is difficult to meet the iron requirements of infants in the absence of fortified foods (23). Iron-fortified formula can play an important role in ensuring adequate iron nutrition for infants (24). However, bacterial contamination of bottle-feeds is a concern (25), and alternative ways of improving the iron intake of infants in developing countries should be sought. High iron-fortified infant-cereals (26), iron drops (27), or iron-enriched sprinkles (28) are the possibilities.

The Global Strategy for Infant and Young Child Feeding acknowledges the role of fortified complementary foods to supply adequate amounts of micronutrients (29). In the study population, it was shown that children who consumed infant-products (infant-cereals, ready-to-eat canned baby-foods and/or formula milk-powder) had significantly higher dietary intakes for most micronutrients (22), and infant-cereals were consumed by more non-anaemic than anaemic infants. Consumption of inadequate quantities of fortified infant-cereals was observed in the study population (22). Fortified infant-cereals are often consumed in a diluted form (5,30), probably because of the cost of these products. Daily consumption of 40 g (dry product) of a specially-formulated low-cost fortified porridge that supplied 11 mg iron per day for six consecutive months was shown to significantly improve the iron status of South African infants (31).

The anaemic infants had a lower dietary intake of animal protein than the non-anaemic infants (Table 3). Addition of 25-g meat to a home-prepared vegetable puree meal of infants aged 7–8 months was shown to increase the absorption of non-heme iron (32), and a small increase in meat intake at the age of eight months prevented a decline in haemoglobin concentration (33). The cost of foods of animal sources may, however, prohibit daily consumption in areas of low socioeconomic status.

Not only was the iron content of the diet lower for the anaemic than for the non-anaemic infants, but the groups also differed in intake of effectors (inhibitors, such as tea, and enhancers, such as vitamin C) of iron bioavailability. Dietary vitamin C intake by the non-anaemic infants was almost twice that of the anaemic infants.

Neonatal weight and length have been shown to be predictors of weight-for-age and length-for-age (34). When controlling for low birth-weight, the anaemic infants were more likely to show growth faltering, both in terms of underweight and stunting. Iron deficiency contributes to poor growth (35), and supplementing anaemic children with iron has a positive effect on linear growth (36). On average, the anaemic infants aged 6-<9 months had a slower growth rate than the non-anaemic infants, suggesting that anaemia restricted child growth, particularly for the younger infant. The anaemic infants did not only have a lower dietary intake of iron compared to the non-anaemic infants, but also of several micronutrients. The low intake of iron certainly contributed towards the association between anaemia and growth faltering, but other micronutrients, such as zinc, could also have played a role as both anaemia and growth faltering often reflect an overall poor nutritional status.

In conclusion, low birth-weight, a mother aged 20 years or younger, and consumption of tea were identified as risk factors for anaemia, while formula feeding had a protective effect. This study further showed that the anaemic infants had a higher chance for growth faltering in terms of both underweight and stunting. The iron content of the diet was lower for the anaemic than for the non-anaemic infants, and the groups differed in intake of several micronutrients and effectors of iron bioavailability. Greater emphasis needs to be given to the nutritional quality of complementary foods.
